# Effectiveness and safety of ferric carboxymaltose compared to iron sucrose in women with iron deficiency anemia: phase IV clinical trials

**DOI:** 10.1186/s12905-017-0506-8

**Published:** 2018-01-05

**Authors:** Amreen Naqash, Rifat Ara, Ghulam N. Bader

**Affiliations:** 1 Department of Pharmaceutical Sciences, University of Kashmir, Srinagar, J&K 190006 India; 2 Department of Obstetrics and Gynecology, SKIMS Medical College and Hospital, Srinagar, J&K 190017 India

**Keywords:** Iron deficiency anemia, Ferric carboxymaltose, Iron sucrose, Hemoglobin, Serum ferritin

## Abstract

**Background:**

Iron deficiency anemia (IDA) is a significant problem worldwide particularly in women. The aim of the study was to evaluate the effectiveness and safety of intravenous ferric carboxymaltose (FCM) in comparison to iron sucrose (IS) in women with IDA.

**Method:**

Two hundred patients at Department of Obstetrics and Gynaecology, *Sher-i*-*Kashmir Institute of Medical Sciences Medical College and Hospital*, Jammu & Kashmir, India identified with IDA were enrolled for the study. Intravenous FCM and IS were both given as per the protocol. Change in the laboratory parameters such as hemoglobin (Hb), mean corpuscular value, and serum ferritin levels at two weeks and four weeks interval after the treatment was recorded.

**Result:**

A significant increase in the mean Hb was observed from 7.76 ± 0.709 to 13.25 ± 0.606 in patients treated with FCM and 7.64 ± 0.710 to 11.59 ± 0.733 g/dL (*P* < 0.001) in patients treated with IS after four weeks of therapy. The rise in mean corpuscular volume was from 66.82 ± 5.24 to 86.76 ± 3.765 and 68.05 ± 5.56 to 93.80 ± 3.80 and rise in serum ferritin levels were from 8.32 ± 1.787 to 38.94 ± 6.095 μg/L and 8.16 ± 1.540 to 27 ± 8.175 μg/L in patients treated with FCM and IS respectively after four weeks of therapy. No serious adverse effects were reported.

**Conclusion:**

Parenteral therapy is effective in IDA, but FCM elevates hemoglobin level and restored iron stores faster than IS with minimum adverse drug reactions.

**Trial registration number:**

ISRCTN14484575 Dated: 15–12-2017 retrospectively registered. https://doi.org/10.1186/ISRCTN14484575

## Background

Anemia, identified by decreased red blood cell count is pathophysiologically multifactorial [1, 2].The World Health Organization (WHO) defines anemia as a hemoglobin (Hb) value below 13 g/dL in men over 15 years of age, below 12 g/dL in non-pregnant women over 15 years, and below 11 g/dL in pregnant women [3]. Half of the world’s anemic burden is contributed alone by iron deficiency anemia [4]. In 2013, 1.2 billion people were found to be affected by iron deficiency anemia (IDA) and caused 183,000 deaths [5]. The condition has an alarming prevalence rate among pregnant women i.e. 88% followed by 74% in non-pregnant women [4].

IDA is caused by depletion of the iron stores which is a consequence of an imbalance between iron uptake and utilization [6]. Iron deficiency is enough to suppress the erythropoiesis resulting in decreased Hb and thus IDA [7].The prevalence of IDA is higher in children and pregnant women, while non-pregnant women and elderly are next. In children, anemia affects the cognitive performance, behavioral and physical development [8, 9], while as in women, anemia affects the productive as well as reproductive abilities which primarily result in poor work capacity, decreased energy, diminished quality of life, fatigue, or even infertility [10]. Anemic state if carried through pregnancy results in retardation of intrauterine growth, increased preterm labor, low birth weight infants, increased chance of perinatal and maternal mortality. The major contributor of IDA in women is low socio-economic status, inadequate diet, and diseased condition [11, 12].

### Evaluation of IDA

Laboratory markers for diagnosis of IDA include complete blood count (CBC) and iron profile (serum iron (SI), serum ferritin (SF), transferrin saturation (TS %) and total iron binding capacity (TIBC)). These parameters are evaluated and accordingly treatment is initiated. Withal, in earlier stages of IDA these laboratory parameters may not help in diagnosis [13–15].The other definitive method for diagnosis of IDA is bone marrow examination for the absence of stainable iron. However, since it is painful and invasive it is not used often and is generally the last resort [16].

### Treatment of IDA

IDA can be reversed by adequate iron replacement therapy, management of the cause of IDA, and maintenance of normal iron levels. The first line therapy: oral therapy, is the intake of 200 mg iron supplement twice or thrice daily [17]. In case oral therapy is ineffective then parenteral therapy becomes the therapy of choice [18]. Parenteral therapy restores the iron stores faster than oral therapy and is well tolerated in pregnancy [19–21]. The most commonly used parenteral preparations are IS and iron dextran [22]. Recently, Food and Drug Administration approved new novel iron formulation ferric carboxymaltose (FCM) is proving to be a better potential for restoring the iron stores. It reduces the dosage frequency which is otherwise a main drawback of parenteral preparations and there are minimal drug related adverse effects [23].

## Method

### Aim

To compare the effectiveness and safety of intravenous ferric carboxymalose (FCM) with iron sucrose (IS) in women with IDA.

### Objectives

To compare the effectiveness and safety of FCM with respect to IS in achieving the target levels of laboratory biomarkers such as Hb, mean corpuscular volume (MCV), serum iron (SI), serum ferritin (SF), transferrin saturation (TS%), total iron binding capacity (TIBC) levels as prescribed under standard guidelines; to identify and prevent any possible drug related problems in patients on FCM or IS; To make suggestions for use of these drugs whenever required by evaluating the laboratory biomarkers; To assess the time required to attain the normal laboratory biomarker levels by these two drugs; and to evaluate health related quality of life (HRQOL) using the Medical Outcomes Study Short Form 36 (SF-36).

### Setting

**Fig. 1:**
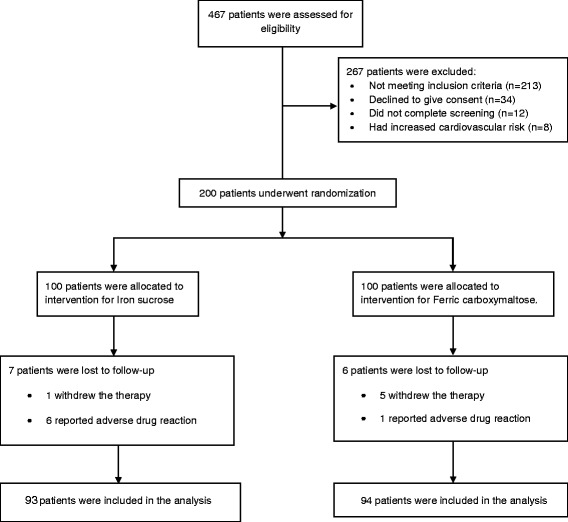
Flowchart of the study

The study was conducted at the Department of Obstetrics and Gynaecology, *Sher-i*-*Kashmir Institute of Medical Sciences* (SKIMS) Medical College and Hospital, Bemina, Srinagar, J&K, India, a tertiary care hospital (which provides specialist care for all age groups) over a period of six months**.** Flow chart of the study is given in Fig. 1. Ethical clearance for the study was obtained from the Institutional Ethics Committee vide clearance certificate no. SKIMS MC/CM/IEC/15/42–50, Dated 16–07-2015. The trial was conducted in accordance with the principles of the Declaration of Helsinki.

### Sample size

Total of 200 patients were enrolled for the study after proper statistical confirmation. Patients were randomly allocated in a 1:1 ratio, to receive either FCM or IS using SNOSE (sequentially numbered, opaque, sealed envelopes). The sample size was kept 100 for each group, so that appropriate statistical tests required for comparative analysis could be employed. This sample size fulfilled the assumptions required for using statistical tests that were used for evaluation of the results.

### Study population

Female patients above 18 years or older with IDA admitted/or those who had come for consultation at the Department of Obstetrics and Gynecology, *Sher-i*-*Kashmir Institute of Medical Sciences* (SKIMS) Medical College and Hospital, Bemina, Srinagar were enrolled for the study and a well-informed written consent was taken from all the patients before starting the therapy.

### Inclusion criteria

All patients diagnosed with IDA admitted/or present for consultation in the Obstetrics and Gynecology ward of SKIMS Medical College and Hospital, Bemina, Srinagar were enrolled during the study period.

### Exclusion criteria

- Patients with uncontrolled hypertension,
- Patients with impaired renal function,
- Patients with impaired liver function,
- Patients with heart disease. [24]

### Data collection

Patient specific and relevant information: Before beginning with the iron correction phase, a clinical history was compiled on a structured data collection form through patient interview. In conformity with the standards given and published in the literature, indicators for drug utilization of FCM and IS were developed, which were the baseline demographic and clinical characteristics of the study patients (Table 1 and Table 2). Hospital doctors and the pharmacologist were involved in choosing a dose, maintenance of the therapy and monitoring of the adverse drug reactions (ADRs). While as, the administration of the drug was done by nursing staff.

**Table 1:** Baseline demographic and clinical characteristics of the study patients

Variable		Iron Sucrose (*N* = 100)	Ferric Carboxymaltose (*N* = 100)
Age – year		27·32 ± 4·15	30·41 ± 7·99
Height – cm		158 ± 6·54	158·29 ± 7·11
Body weight – kg		59·07 ± 6·91	59·36 ± 6·23
Body mass index^a^		23·68 ± 2·56	23·80 ± 3·10
Blood Pressure – mm Hg	Systolic	111·86 ± 6·68	110·85 ± 7·10
	Diastolic	71·71 ± 5·39	71·57 ± 5·19
Pulse – beats/min		80·49 ± 4·57	81·43 ± 5·46
Medical Condition –– no.			
Pregnancy			
First Trimester		–	–
Second Trimester		8	9
Third Trimester		39	39
Postpartum		20	19
Menorrhagia^b^		20	18
Fibroid Uterus + Heavy Bleeding^c^		6	7
Ovarian cyst		7	7
Ligation		–	1
Co-morbidities –– no.			
Diabetes		2	3
Thyroid Disorder		6	8
Gastrointestinal Disorder		9	13
Urinary tract infections		7	9
Vitamin and Mineral Deficiencies		47	45
Other		12	11
Concomitant Treatment –– no.			
Insulin		2	3
Drugs for Thyroid Disorder		6	8
Antacids/ PPIs		11	11
Antibiotics		11	18
Multi-vitamin and Mineral supplements.		49	48
Other		9	10

± Plus-minus values are of standard deviation

^a^The body mass index is the weight in kilograms divided by the height in meters

^b^Bleeding have been continuous for more than 7 days with heavy clots, disrupted cycle associated with pain. More than 2 pads or tampons every 2 h, doubling of pads or tampons to manage menstrual flow

^c^Prolonged periods (>12 days); hot flushes; disrupted sleep pattern; pain in joints; More than 2 pads or tampons every 1 h, doubling of pads or tampons to manage menstrual flow

**Table 2:** Baseline clinical data

Laboratory Parameters	Iron Sucrose	Ferric Carboxymaltose	*p* value
Haemoglobin –– g/dL	7·64 ± 0·72	7·82 ± 0·75	0·0850
Mean Corpuscular Volume –– fL	66·82 ± 5·24	68·05 ± 5·56	0·1106
Serum Iron –– μg/dL	32·75 ± 6·80	34·46 ± 7·38	0·0901
Serum Ferritin –– μg/dL	18·13 ± 1·67	18·29 ± 2·16	0·5589
Total Iron Binding Capacity –– μg/dL	474·18 ± 43·76	457·94 ± 66·96	0·0436
Transferrin Saturation –– %	7·02 ± 1·611	7·67 ± 1·97	0·118

± Plus-minus values are of standard deviation

### Protocol for evaluation and treatment

The total iron required for iron repletion was calculated at baseline, according to the Ganzoni’s Formula [25] *Total iron dose = {(Body weight) [kg] × (Target Hb -Actual Hb) [g/L]} × 0*.*24 + Iron stores [mg]* where, 0.24 is a correction factor that takes into account the patient’s blood volume, estimated at 7% of body weight and Hb iron content; which is 0.34%.

Intravenous (IV) iron infusion was given according to the iron deficit calculated by and rounded up to the nearest multiple of 100 for each individual [19]. Before starting the infusion, due to the limited availability of safety data for use of FCM in pregnancy, a test dose was given to check for any adverse reaction for both, FCM and IS [26]. In case of IS, the maximum dose of 200 mg was diluted in 200 ml of sterile normal saline 0.9% and was given as slow infusion over 30 min. The rest of the doses, as and when required, were given on alternate days following the same procedure [27]. For FCM, the maximum single dose of 1000 mg diluted in 250 ml of sterile normal saline 0.9% was given as slow infusion over 45 min. If needed, rest of the doses were given on the 8th and the 15th day [27, 28]. In case of any adverse drug reaction (ADR), the infusion was stopped, documented, and the patient was treated for the respective ADR. After the treatment, the patient’s progress towards accomplishment of the goal of therapy was evaluated and the outcomes were analyzed. The well-being of the patients was evaluated through a questionnaire for health-related quality of life (HRQOL). The Medical Outcomes Study Short Form 36 (SF-36) is a popular HRQOL measure. SF-36 provides a measure of eight health-related domains, such as physical functioning, role limitations due to physical health, role limitations due to emotional problems, emotional well-being, social functioning, energy/fatigue, Pain and general health perceptions. This HRQOL measure is obtained through patient interview and covers questions related to both mental and physical aspects of health [29]. Follow up of patients in both groups was done at 2 and 4 weeks interval from the baseline. The patients were also counselled by the clinicians for having iron rich diet and animal protein foods (meat, fish and poultry) which enhance iron absorption. The end point was taken as attainment of normal Hb, MCV, SI, SF, TIBC, TS%.

## Statistical analysis

The baseline changes and the values at weeks 2 and 4 for continuous variables were compared between the FCM and IS groups. Available pre-infusion, post-2 week infusion and 3 week infusion Hb, MCV, SI, SF, TIBC and TS% levels were compared using one-way ANOVA tests. Also, the statistical analysis of HRQOL parameters were obtained from a comparison using the unpaired t-test. Repeated measure analysis was carried out to see the trend of parameters with time. To compare efficacy of the two drugs Z-test was used. Confidence intervals (95%) of various populations were also calculated. *p < 0*.*005* was taken as significant. The statistical analysis was done using SPSS version 20 software.

## Result

**Fig. 2:**
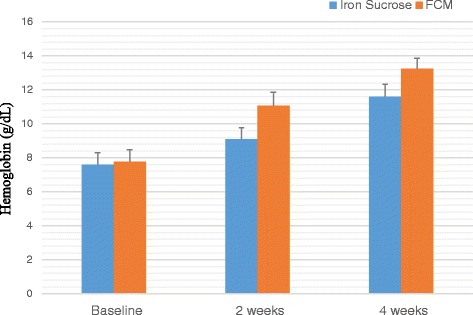
Comparison of hemoglobin (μg/dL) levels before and after IS and FCM therapy

**Fig. 3:**
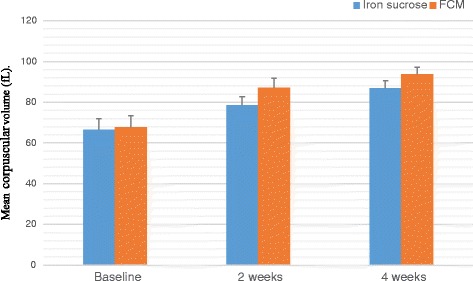
Comparison between mean corpuscular volume (fL) levels before and after IS and FCM therapy

**Fig. 4:**
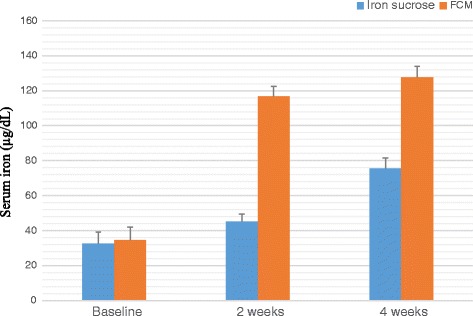
Comparison between serum iron (μg/dL) levels before and after IS and FCM therapy

**Fig. 5:**
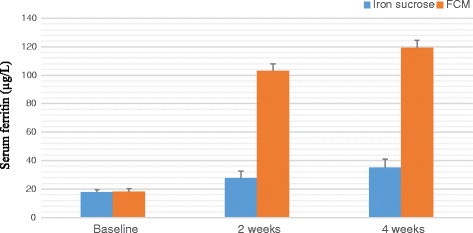
Comparison of serum ferritin (μg/L) levels before and after IS and FCM therapy

**Fig. 6:**
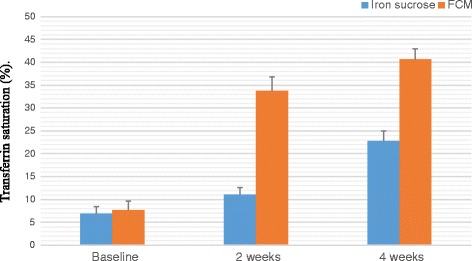
Comparison between transferrin saturation (%) levels before and after IS and FCM therapy

**Fig. 7:**
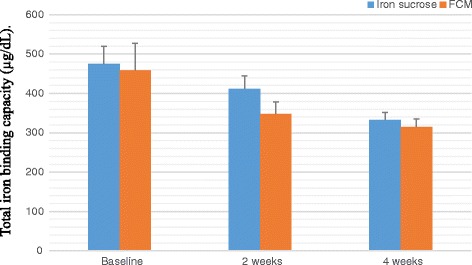
Comparison between total iron binding capacity levels before and after IS and FCM therapy

**Fig. 8:**
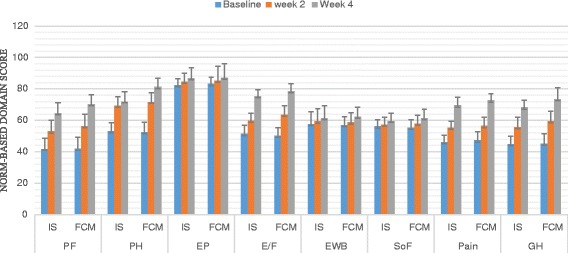
HRQOL mean score (with 95% Confidence Interval) for IS and FCM therapy for the physical functioning (PF), role limitations due to physical health (PH), role limitations due to emotional problems (EP), Energy/Fatigue (E/F), emotional well-being (EWB), social functioning (SoF), pain, general health (GH) domains at baseline, week 2 and week 4

A total of 100 patients were counselled for an iron rich diet and enrolled for each FCM and IS treatment. The clinical characteristics of 200 patients are presented in Table 1 and Table 2. The demographic data like age, body mass index, habitat, type of risk factors were comparable among the two groups. The groups were similar with respect to the baseline clinical, laboratory and HRQOL characteristics at the time of enrolment. Baseline Hb, MCV, SI, SF, TIBC, TS% levels and HRQOL characteristics in the two groups were clinically insignificant. There was an overall increase in laboratory values from baseline at 2 weeks and 4 weeks which is significant between the groups as well as within the groups. Regarding measures of efficacy, the patients that received FCM reported significantly higher laboratory values at 4th week (Hb, MCV, SI, SF, TIBC, and TS% levels) than patients who received IS (*p < 0.0001* for all comparisons) (Figs. 2, 3, 4, 5, 6 and 7). The mean Hb at 4th week was recorded as 13.25 ± 0.606 in FCM group and 11.59 ± 0.733 in IS group (*p < 0.0001*). This proves FCM better as compared to IS in IDA management. Both FCM and IS showed increase in serum ferritin levels, but FCM showed significant increase in the ferritin levels. Table 3 gives the details of mean value of other laboratory parameters, proving FCM to be a better therapy in IDA than IS. The HRQOL score based on SF-36 for each of the eight health domains are shown in Fig. 8. At baseline no significant difference between patients treated with IS or FCM was noted. However, after treatment HRQOL score based on SF-36 increased in both groups, but was better in patients treated with FCM as compared to IS treated patients. Domains like physical functioning, role limitations due to physical health, energy/fatigue, pain, and general health showed statistically significant (*p < 0.005)* difference between two groups at 4th week. Whereas, role limitations due to emotional problems, emotional well-being, and social functioning were statistically insignificant at 4th week.

**Table 3:** 4^th^ Week clinical data

Laboratory Parameters	Iron Sucrose *N* = 93	Ferric Carboxymaltose *N* = 94	*p* value
Haemoglobin –– g/dL	11·59 ± 0·733	13·25 ± 0·606	0·0001
Mean Corpuscular Volume –– fL	86·76 ± 3·765	93·80 ± 3·380	0·0001
Serum Iron –– μg/dL	75·40 ± 6·223	127·54 ± 6·321	0·0001
Serum Ferritin –– μg/dL	34·99 ± 6·250	119·02 ± 5·487	0·0001
Total Iron Binding Capacity –– μg/dL	332·61 ± 18·619	315·13 ± 19·981	0·0001
Transferrin Saturation –– %	22·76 ± 2·183	40·59 ± 2·321	0·0001

± Plus-minus values are of standard deviation

## Follow up

Out of the 100 patients that were enrolled for the study of FCM, six (6%) did not complete the 4 week follow up; of these six, five patients withdrew therapy and one reported ADR. Similarly, out of the 100 patients that were enrolled for the study of IS, seven (7%) did not complete the 4 week follow up; of these seven, one patient withdrew therapy and 6 reported ADR.

### Adverse drug effect

Three patients complained of nausea and tingling sensation, two complained of headache after the sixth dose of IS. One patient complained of arthralgia at the start of the seventh dose of IS, while one patient complained of headache after the second dose of FCM. However, no serious ADR was reported in any patient.

### Patient compliance

Patients who were treated with FCM reported satisfaction and better HRQOL (Fig. 8). Also, patients treated with FCM had low dose frequency which decreased the number of visits to the hospital and in turn resulted in exhausting minimum hospital resources and increase in acceptability as compared to patients treated with IS.

## Discussion

The aim of the study was to compare the safety and efficacy of FCM with IS in women with IDA. Since the population under study was not homogenous, the main causes of IDA were diverse such as a menorrhagia, pregnancy to postpartum anemia with co-morbidities like diabetes, inflammatory bowel disease, thyroid disorder, urinary tract infections, and other mineral or vitamin deficiencies.

The patient population was identified on the basis of laboratory biomarkers— Hb, MCV, SI, SF, TS%, TIBC. These variables were also used to calculate the iron deficit and iron repletion dose. Also, decision regarding the continuation of therapy or interruption was taken on the basis of these laboratory biomarkers.

A treatment with FCM for 4 weeks in patients with IDA improved laboratory biomarkers (Hb, MCV, SI, SF, TS%, TIBC), symptoms, and the HRQOL score in a shorter duration of time as compared to IS.

The study also shows that treatment with FCM was not associated with any unacceptable side effects or adverse effects. Only one patient complained of a mild headache after IV dose of FCM as compared to 6 patients (in IS recipients) who suffered from nausea, tingling sensation, headache, and arthralgia. Overall, both drugs did not show any serious ADR and these are expected events that are reported in previous literature [27, 28, 30, 31]. The study is consistent with other reported comparative studies with IS or other preparations.

In a randomized trial, patients treated with FCM achieved Hb rise of >2 g/dL in 7 days and >3 g/dL in 2–4 weeks as reported by Van Wyck and colleagues [32].In our study the mean increase after 2 weeks was recorded as 3.32 g/dL after 2 weeks and 4.92 g/dL after 4 weeks from baseline value in case of patients treated with FCM.

In a study by Giannoulis and colleagues [33], the increase in Hb was 4–6 g/dL in 4 weeks in patients receiving IS, whereas in our study the Hb levels showed 3.9 g/dL increase after 4 weeks.

In the study of FCM by Seid and colleagues [31], the rise in Hb was 3 g/dL or more, in 15 days which is in consistence with our study.

Breymann and colleagues [30] reported the increase in ferritin levels from 39.9–150 μg/L at 4th week. In our study, we observed in FCM group, mean ferritin level increased from baseline value of 18.28–119.02 μg/L at 4th week.

Adverse events reported under various studies are between 6.8% and 24.2% [30, 31] for IS and in our study it was 6%. None of the patients reported any serious ADR requiring hospitalization. Aggarwal and colleagues [34] reported fever, arthritis, dyspepsia, and anaphylaxis Grade I in patients receiving IS therapy, whereas in our study adverse events were mild headache, nausea, arthralgia, and tingling sensation. Also, FCM was better tolerated in pregnant women with no reported ADRs which is in consistence with the study by Breymann and colleagues [35].

Favrat and colleagues [36] have reported the improved HRQOL score in patients treated with IV FCM which is in consistence with our study.

On the basis of the current study, both FCM and IS are safe and efficacious. However, FCM shows significant attainment of ferritin and Hb levels after 4 weeks than IS.

## Strength of the study

This is the first study assigning the comparison of two IV preparations in the state of Jammu and Kashmir. Limited data is available with respect to comparison of safety and efficacy of IS and FCM. This study gives an insight about the adverse effects and the data is reliable as it was progressively collected and documented during and after the treatment. Patients were monitored 30 min after the infusion to ensure keen observation and maximum safety.

## Limitation of the study

The limited number of patients (population understudy were only females) and less time duration are the limitations of this study. Also, in case of pregnant women no data regarding neonatal outcome was recorded. The study needs to be done on a larger population (both male and female and all age groups) and the time duration needs to be more to assess the long term effects of these drugs on other biomarkers like electrolytes, C-reactive protein, etc. Comparison also needs to be done with other preparations available in the market.

## Conclusion

Iron deficiency anemia is widespread and mostly overlooked. Prophylactic treatment should be started in women with heavy menstrual bleeding and pregnancy to avoid any iron deficiency related complications. This study in conclusion demonstrates that a single dose of 1000 mg IV FCM infusion is safe and effective in the treatment of IDA in women. FCM improves laboratory biomarkers i.e., Hb, MCV, SI, SF, TIBC, TS% levels and restores the iron stores in short duration of time as compared to IS. The patient compliance is better with FCM as compared to IS as FCM reduces the number of hospital visits. FCM is ideally suited for treatment of patients with IDA who require rapid replenishment of iron stores.

## Abbreviations

ADR: Adverse Drug Reaction
CBC: Complete blood count
FCM: Ferric Carboxymaltose
Hb: Hemoglobin
HRQOL: Health related quality of life
IDA: Iron Deficiency Anemia
IS: Iron Sucrose
IV: Intravenous
MCV: Mean Corpuscular Volume
SF: Serum Ferritin
SF-36: Study-Short Form 36
SNOSE: Sequentially numbered, opaque, sealed envelopes
TIBC: Total Iron Binding Capacity
TS%: Transferrin saturation
